# Comparative efficacy of interventions for jaundice in hilar cholangiocarcinoma: A retrospective analysis using the Mayo staging system

**DOI:** 10.1097/MD.0000000000049614

**Published:** 2026-07-03

**Authors:** Zepu Wang, Dan Lv, Hanxiang Yu, Chuncheng Wang, Yanhui Peng

**Affiliations:** a Department of Hepatobiliary and Pancreatic Surgery II, Hebei General Hospital, Shijiazhuang, Hebei, China; b Ultrasound Department, Hebei General Hospital, Shijiazhuang, Hebei, China.

**Keywords:** EBD, hilar cholangiocarcinoma, Mayo staging, median survival, PTBD

## Abstract

This study aimed to evaluate the clinical efficacy of 3 jaundice reduction methods in patients with intermediate or advanced hilar cholangiocarcinoma (HCCA) at different Mayo stages. Clinical data from 223 patients with HCCA who underwent palliative jaundice reduction treatment were collected and analyzed retrospectively. A statistical analysis was conducted to compare the therapeutic effects, survival times, complications, and hospital stays of patients in the 3 groups with different Mayo stages. Survival curves were plotted for patients with different Mayo stages. There was no statistically significant difference in decreased liver enzyme and bilirubin levels among the 3 patient groups after surgery (*P* > .05). For patients at Mayo stages I and II, the median survival time for those who underwent palliative surgery was longer than for those who underwent percutaneous transhepatic biliary drainage (PTBD) or endoscopic biliary drainage (EBD) (*P* = .001 and *P* < .001, respectively). For patients at Mayo stages III and IV, however, there was no statistically significant difference in median survival time between the 3 groups (*P* > .05). There was also no statistically significant difference in the incidence of complications in each group at every stage (*P* > .05). Hospital stays for the palliative surgery group were significantly longer than for the PTBD and EBD groups (*P* < .001). For HCCA patients in Mayo stages I and II, palliative surgical treatment should be prioritized. For patients at Mayo stage III, EBD treatment is more appropriate. For patients in Mayo stage IV with poor general health, PTBD is a more appropriate choice.

## 1. Introduction

Hilar cholangiocarcinoma (HCCA) is an aggressive form of cancer that originates in the bile duct. It accounts for 60 to 70% of all cholangiocarcinomas. It has a poor prognosis, with a 5-year survival rate of just 5 to 15%.^[1,2]^ Although radical resection surgery is the main curative treatment for HCCA, most patients have lost the opportunity for this type of surgery by the time they seek medical attention, due to the tumor’s concealed location and lack of early symptoms. Only 10 to 20% of patients are eligible for radical surgery, with the majority receiving palliative care instead.^[3,4]^ Effective bile drainage can relieve jaundice and promote the excretion of metabolic waste products and toxins. It reduces liver cell damage and ultimately improves quality of life and prolongs the survival period.^[5,6]^ Currently, the 3 most common palliative methods for reducing jaundice are palliative surgery, percutaneous transhepatic biliary drainage (PTBD), and endoscopic biliary drainage (EBD). Each of these methods has its own advantages and disadvantages. While some scholars have evaluated the effectiveness of PTBD and EBD in treating malignant obstructive jaundice, no consensus has yet been reached.^[7,8]^ Currently, there are no specific studies on the most effective way to reduce jaundice in patients with intermediate or advanced HCCA.

The Mayo staging system, developed by Chaiteerakij et al in 2014, is based on an analysis of the clinical data of 413 patients with HCCA who were treated at the Mayo Clinic in the United States. This staging system divides patients into four stages based on the Eastern Cooperative Oncology Group (ECOG) score, carbohydrate antigen 199 level, tumor size and number, tumor and lymph node metastasis, and vascular involvement.^[9]^ As Mayo staging does not rely on pathological information, it is particularly suitable for assessing the prognosis of patients who cannot undergo surgical resection. Studies have shown that the Mayo staging system is more effective in predicting survival.^[10,11]^

The present study stratifies patients based on the Mayo stage to conduct a comparative analysis of 3 jaundice reduction methods for patients with advanced HCCA at different Mayo stages, evaluating their safety and clinical efficacy. The objective of this study is to establish the most effective and optimal palliative jaundice reduction treatment strategy for patients with advanced HCCA, thereby providing a foundation for the development of customized treatment plans in clinical practice.

## 2. Materials and methods

### 2.1. Patients

The clinical data of 223 patients diagnosed with HCCA who received palliative jaundice reduction treatment at Hebei General Hospital from January 2016 to January 2024 were collected and retrospectively analyzed. The subjects were divided into 3 groups according to the jaundice reduction methods: the palliative surgery group, with 80 patients; the PTBD group, with 60 patients; and the EBD group, with 60 patients. The stratification process is conducted in accordance with the Mayo Staging System.^[9]^ The inclusion criteria are as follows: the subject must be aged 18 years or over; a clear diagnosis of HCCA is required, made on the basis of clinical manifestations, laboratory tests, imaging tests, or biopsy. The following criteria were used to determine exclusion from the study: benign obstructive jaundice; gallbladder cancer, gallbladder duct cancer; distal cholangiocarcinoma; receipt of radical surgical resection; incomplete data. The study was approved by the Ethics Committee of Hebei General Hospital (No. 2021-157), conducted per the Declaration of Helsinki, and the committee waived the requirement for written informed consent due to the retrospective data and concealment of patient identifiers.

### 2.2. Methods of operation

Palliative surgery: After opening the abdomen, the location of tumor invasion was explored, and choledocho-jejunal Roux-en-Y anastomosis was performed. The liver was pulled upwards to reveal the hepatoduodenal ligament. The extrahepatic bile ducts and the gallbladder were resected to the maximum extent possible, and the distal choledochal ducts were closed by suture stump. The proximal left and right hepatic ducts were freed for anastomosis. The jejunum and jejunal mesentery were cut off at 10 to 15 cm from the duodenojejunal flexure. The 2 arteries of the first-stage arterial arch of the jejunal mesentery were cut off, and the distal end of the jejunum was poked through a hole in the transverse colonic mesentery and lifted up to the porta hepatis, and then anastomosed with biliary anastomosis. The proximal jejunum was laterally anastomosed to the jejunum about 45 to 50 cm distal to the biliary anastomosis. The anastomosis was then closed with sutures.

PTBD: Take the supine position. Under ultrasound or X-ray monitoring, determine the needle point and needle angle according to the location of the bile duct. Then, puncture into the skin with an 18 to 21G plastic trocar under aseptic operation to quickly enter the bile duct through the liver and leave external drainage. If internal stent placement is performed, after the puncture needle enters the intrahepatic bile duct, the metal guide wire passes through the obstructed segment to reach the duodenum. The extended guide wire is placed into the internal stent. Then, after imaging confirms that the internal stent has crossed the stenotic segment and the bile duct is clear, the external biliary drainage tube is left in order to prevent the stent from being occluded.

EBD: In the left prone position, endoscopic retrograde cholangiopancreatography was performed with a duodenoscope. After the site, degree, and scope of the obstruction were clarified by imaging, the guide wire was placed into the bile duct and over the site of the obstruction. The stenosis was dilated by biliary dilatation probes, and then a metal stent of 6 to 8 cm in length or a plastic stent of 8.5 to 10 Fr was placed, both ends of which exceeded the obstructed segment by more than 1 cm.

### 2.3. Data collection and follow-up

The collected data includes age and gender, the number and diameter of tumors, vascular invasion and metastasis, ECOG score, relevant laboratory indicators before and 7 days after the operation, occurrence of complications, hospital stay, etc. Follow-up visits mainly consist of telephone and outpatient consultations. Patients are followed up once a month. Survival is defined as the period from the date of surgery to the date of death or the last follow-up.

### 2.4. Statistical analysis

SPSS 21.0 software was used for the statistical analysis. Normally distributed measurements were expressed as mean ± standard deviation and compared using analysis of variance. Non-normally distributed measurements were expressed as median (P25, P75) and compared using the Kruskal–Wallis H test. Count data were expressed as numbers or percentages and compared using the *X*^2^ test. The survival times of groups with different Mayo stages were analyzed using the Kaplan–Meier method. A multivariable Cox regression model was used to identify independent prognostic factors, and *P* < .05 was considered statistically significant.

## 3. Results

### 3.1. Comparison of general data for the 3 patient groups

Following statistical analysis, no significant differences were found in terms of age, gender ratio, proportion of Mayo stage, ECOG score, proportion of adjuvant therapy, preoperative total bilirubin (TBil) or hepatic enzyme level, or preoperative carbohydrate antigen 199 level between the palliative surgery, PTBD, and EBD groups (*P* > .05). The data for the 3 groups were comparable (Table 1).

**Table 1:** Comparison of baseline data between the 3 groups.

	Palliative surgery (n = 80)	PTBD (n = 60)	EBD (n = 83)	*P*
Gender, n (%)				
Male	49 (61.30%)	37 (61.70%)	55 (66.30%)	.768
Female	31 (38.80%)	23 (38.30%)	28 (33.70%)	
Age (yrs)	67.95 ± 8.76	70.38 ± 10.73	69.29 ± 11.55	.385
Mayo stage, n (%)				
I	23 (28.75%)	13 (21.67%)	21 (25.30%)	.740
II	24 (30.00%)	14 (23.33%)	19 (22.89%)	
III	19 (23.75%)	17 (28.33%)	22 (26.51%)	
IV	14 (17.50%)	16 (26.67%)	21 (25.30%)	
ECOG score, n (%)				
0	31 (38.75%)	28 (46.67%)	38 (45.78%)	.419
1	43 (53.75%)	31 (51.67%)	29 (34.94%)	
2	4 (5.00%)	0 (0.00%)	10 (12.05%)	
3	2 (2.50%)	1 (1.67%)	6 (7.23%)	
Pretreatment TBil (umol/L)	165.10 (75.65–358.00)	252.60 (146.10–337.13)	177.80 (119.00–333.90)	.164
Pretreatment Direct bilirubin (umol/L)	88.80 (38.05–194.83)	133.45 (80.25–173.90)	103.40 (64.50–186.50)	.110
Pretreatment ALT(U/L)	148.00 (78.00–257.00)	115.00 (63.00–205.00)	117.00 (63.00–231.00)	.174
Pretreatment AST (U/L)	121.00 (70.00–200.00)	141.00 (79.00–214.00)	108.00 (68.00–207.00)	.542
Pretreatment GGT (U/L)	640.00 (333.00–1084.00)	462.00 (292.00–1016.00)	728.00 (435.00–997.00)	.197
Pretreatment ALP (U/L)	394.00 (245.00–771.00)	526.00 (310.00–928.00)	539.00 (384.00–762.00)	.108
Pretreatment CA199 (U/mL)	191.27 (87.50–1000.00)	409.65 (110.80–1000.00)	452.70 (111.50–1000.00)	.101
Adjuvant therapy, n (%)				
Yes	52 (65.00%)	31 (51.70%)	47 (56.60%)	.265
No	28 (35.00%)	29 (48.30%)	36 (43.40%)	

ALP = alkaline phosphatase, ALT = alanine aminotransferase, AST = aspartate aminotransferase, CA199 = carbohydrate antigen 199, EBD = endoscopic biliary drainage, ECOG = Eastern Cooperative Oncology Group, GGT = gamma-glutamyl transferase, n = number of participants, PTBD = percutaneous transhepatic biliary drainage, TBil = total bilirubin.

### 3.2. Comparison of therapeutic effects

A comparison and analysis of the decline in liver enzyme and bilirubin levels in the 3 patient groups after surgery revealed significant decreases in TBil, direct bilirubin, alanine aminotransferase, aspartate aminotransferase, gamma-glutamyl transferase, and alkaline phosphatase. However, there was no statistically significant difference in the extent of the decline between the groups (*P* > .05) (Table 2).

**Table 2:** Comparison of the reduction in bilirubin and liver enzyme levels among the 3 groups.

	Palliative surgery (n = 80)	PTBD (n = 60)	EBD (n = 83)	*P*
TBil alleviation (umol/L)	108.10 (45.20–290.03)	111.90 (77.28–149.00)	88.50 (46.50–160.20)	.279
Q1	32.90 (9.25–55.30)	68.00 (46.90–78.35)	15.75 (6.48–42.08)	.004
Q2	91.40 (70.43–116.80)	104.70 (78.10–126.00)	79.30 (62.00–109.45)	.274
Q3	187.00 (128.00–234.20)	137.50 (94.60–191.83)	132.50 (94.50–170.30)	.099
Q4	385.75 (294.13–491.80)	133.35 (107.48–205.88)	229.10 (99.20–326.70)	< .001
Direct bilirubin alleviation (umol/L)	51.20 (25.03–154.30)	62.75 (41.80–102.13)	63.90 (25.80–107.60)	.855
ALT alleviation (U/L)	81.00 (37.00–203.00)	70.00 (21.00–140.00)	64.00 (18.00–155.00)	.122
AST alleviation (U/L)	70.00 (26.00–144.00)	85.00 (23.00–158.00)	60.00 (22.00–144.00)	.801
GGT alleviation (U/L)	418.00 (185.00–727.00)	244.00 (119.00–672.00)	324.00 (176.00–629.00)	.133
ALP alleviation (U/L)	205.00 (98.00–545.00)	248.00 (94.00–522.00)	176.00 (91.00–341.00)	.455

ALP = alkaline phosphatase, ALT = alanine aminotransferase, AST = aspartate aminotransferase, EBD = endoscopic biliary drainage, GGT = gamma-glutamyl transferase, n = number of participants, PTBD = percutaneous transhepatic biliary drainage, TBil = total bilirubin.

**Figure 1.**
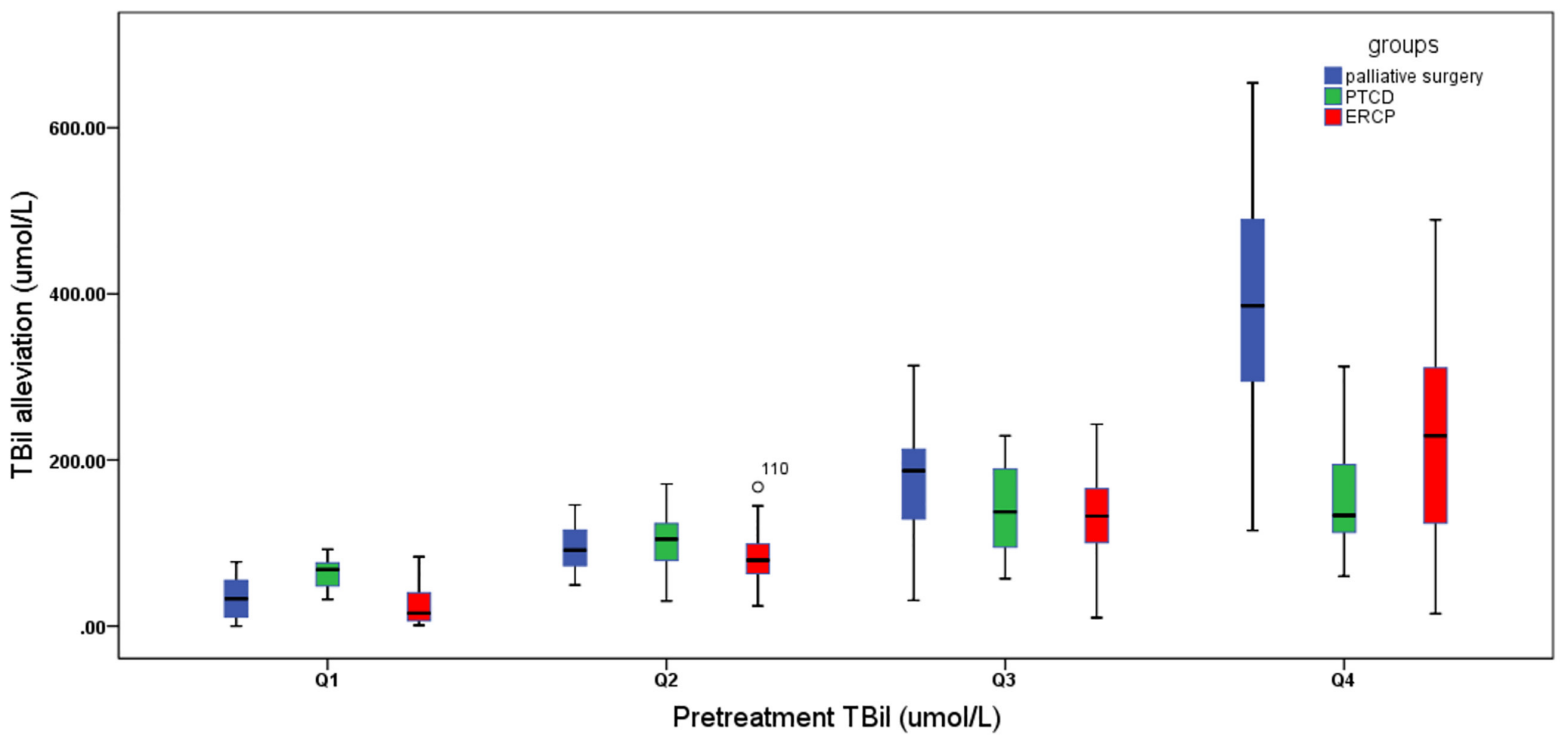
Comparison of TBil reduction among groups stratified by preoperative TBil quartiles. Comparison of TBil reduction among palliative surgery, PTBD, and EBD groups, stratified by preoperative TBil quartiles (Q1–Q4). Data are shown as mean ± SD. EBD = endoscopic biliary drainage, PTBD = percutaneous transhepatic biliary drainage, SD = standard deviation, TBil = total bilirubin.

To further evaluate the reduction of TBil, patients were stratified into quartiles (Q1–Q4) based on preoperative TBil levels. The comparison across treatments revealed that PTBD was superior to the other 2 methods in the Q1 subgroup (*P* = .004). In contrast, palliative surgery yielded the greatest TBil reduction in the Q4 subgroup (*P* < .001). No significant differences were found among the 3 treatments in the Q2 and Q3 subgroups (*P* > .05) (Table 2, Fig. 1).

### 3.3. Comparison of survival times

**Figure 2.**
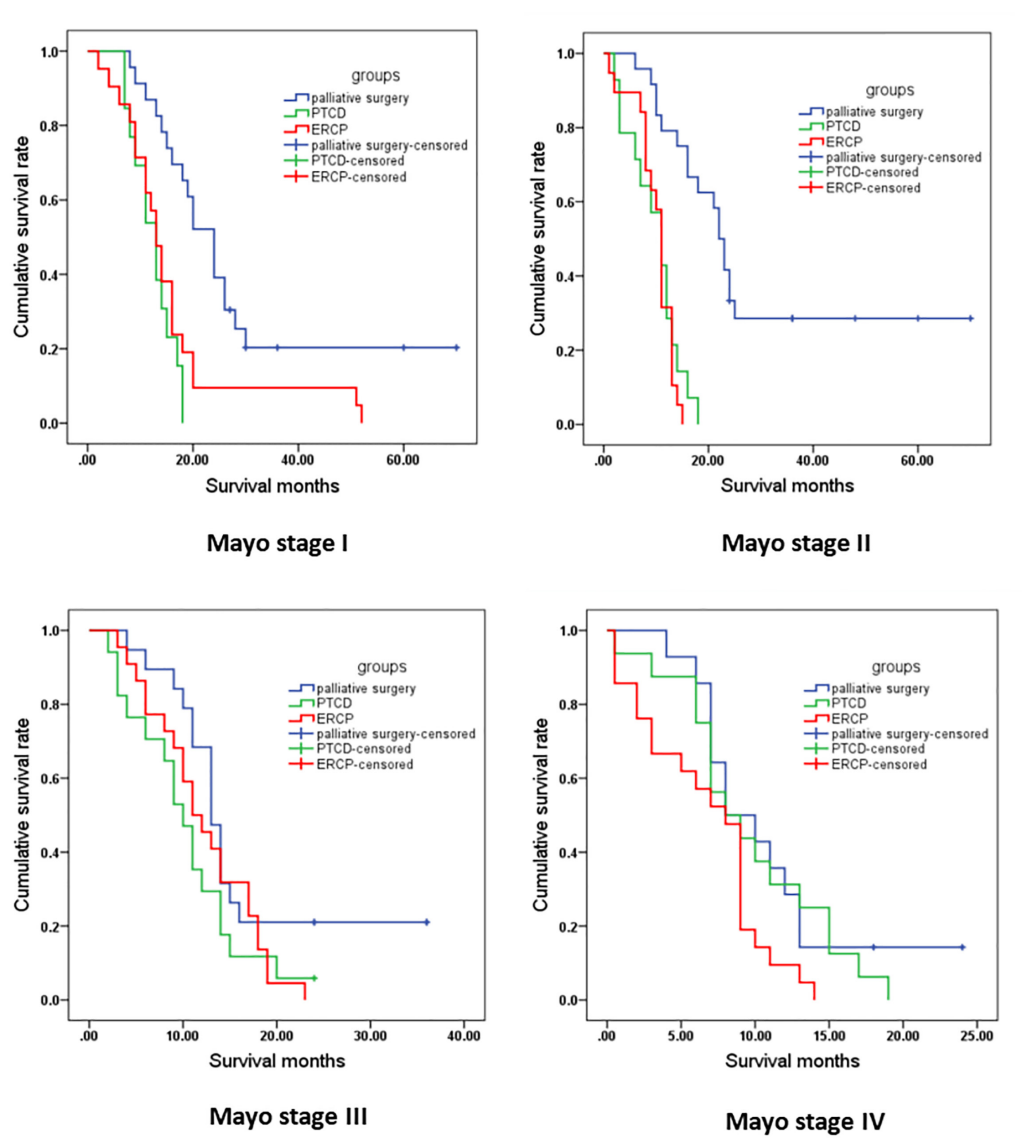
Kaplan–Meier survival curves stratified by Mayo stage. Kaplan–Meier survival curves of patients treated with palliative surgery, PTBD, or EBD, stratified by Mayo stage (I/II, III, IV). EBD = endoscopic biliary drainage, PTBD = percutaneous transhepatic biliary drainage.

Patients were stratified according to the Mayo staging system, and survival times were compared between the 3 respective groups at each stage. The results showed that the median survival time for patients in Mayo stages I and II who underwent palliative surgery was longer than for those who underwent PTBD or EBD, and these differences were statistically significant (*P* = .001 and *P* < .001, respectively). For patients in Mayo stages III and IV, however, there was no statistically significant difference in median survival time between the 3 groups (*P* = .119 and *P* = .131, respectively) (Table 3). Figure 2 shows the survival curves of the 3 patient groups with different Mayo stages.

**Table 3:** Comparison of survival time in 3 groups of patients with different Mayo stages (months).

	Palliative surgery	PTBD	EBD	*P*
Mayo stage I	24.00 (15.00–28.00)	13.00 (8.50–16.00)	13.00 (9.00–17.00)	.001
Mayo stage II	22.50 (14.50–33.25)	11.00 (5.25–13.25)	11.00 (8.00–13.00)	< .001
Mayo stage III	13.00 (11.00–16.00)	10.00 (5.00–14.00)	11.50 (7.50–17.25)	.119
Mayo stage IV	9.00 (7.00–13.00)	8.50 (6.25–14.50)	8.00 (2.50–9.00)	.131

EBD = endoscopic biliary drainage, PTBD = percutaneous transhepatic biliary drainage.

### 3.4. Multivariable Cox regression analysis

A multivariable Cox regression model was used to determine the independent prognostic value of the treatment modality, adjusted for age, pretreatment TBil, ECOG score, and Mayo stage. The treatment method remained an independent predictor of overall survival. The risk of mortality was significantly higher with PTBD (hazard ratio = 3.302, 95% confidence interval 2.209–4.936, *P* < .001) and EBD (hazard ratio = 3.144, 95% confidence interval 2.170–4.554, *P* < .001) than with palliative surgery. Poorer ECOG scores and advanced Mayo stages were also identified as independent risk factors (Table 4).

**Table 4:** Factors associated with overall survival in the multivariable Cox proportional hazards model.

Variable	HR	95% CI	*P*
Age	1.048	1.029–1.068	< .001
Pretreatment TBil	1.000	0.999–1.001	.504
Treatment group			
Palliative surgery	1	-	-
PTBD	3.302	2.209–4.936	< .001
EBD	3.144	2.170–4.554	< .001
Mayo stage			
I	1	-	-
II	0.524	0.290–0.947	.032
III	1.033	0.647–1.650	.891
IV	1.929	1.136–3.275	.015
ECOG score			
0	1	-	-
1	1.798	1.149–2.816	.010
2	2.576	1.232–5.388	.012
3	3.468	1.401–8.584	.007

CI = confidence interval, EBD = endoscopic biliary drainage, ECOG = Eastern Cooperative Oncology Group, HR = hazard ratio, PTBD = percutaneous transhepatic biliary drainage, TBil = total bilirubin.

### 3.5. Comparison of complication rates

A total of 62 complications occurred in the 3 patient groups after surgery. Of these, 28 cases occurred in the palliative surgery group and were mainly due to hypoproteinemia, anemia, deep vein thrombosis, bile leakage, electrolyte disorders, infections, and bleeding. Sixteen cases occurred in the PTBD group, primarily due to electrolyte disorders, biliary tract infections, and biliary tract bleeding. Eighteen cases occurred in the EBD group, primarily due to acute pancreatitis, biliary tract infections and bleeding, and electrolyte disorders. While the palliative surgery group had a higher number and variety of complications than the PTBD and EBD groups, the differences in complication rates among the 3 groups were not statistically significant after stratification according to Mayo staging (*P* = .365, 0.391, 0.562 and 0.781, respectively) (Table 5).

**Table 5:** Comparison of complications in 3 groups of patients with different Mayo stages.

	Palliative surgery	PTBD	EBD	*P* value
Mayo stage I	7/23 (30.43%)	2/13 (15.38%)	3/21 (14.29%)	.365
Mayo stage II	8/24 (33.33%)	3/14 (21.43%)	3/19 (15.79%)	.391
Mayo stage III	7/19 (36.84%)	6/17 (35.29%)	5/22 (22.73%)	.562
Mayo stage IV	6/14 (42.86%)	5/16 (31.25%)	7/21 (33.33%)	.781

EBD = endoscopic biliary drainage, PTBD = percutaneous transhepatic biliary drainage.

### 3.6. Comparison of length of stay

Patients in the palliative surgery group had a significantly longer length of stay at each Mayo stage than those in the PTBD and EBD groups (*P* < .001). However, no significant difference in length of stay was observed between the PTBD and EBD groups (*P* > .05) (Table 6).

**Table 6:** Comparison of length of stay in 3 groups with different Mayo stages (days).

	Palliative surgery	PTBD	EBD	*P* value
Mayo stage I	22.43 ± 4.82	10.08 ± 2.93	10.95 ± 3.25	< .001
Mayo stage II	22.38 ± 4.53	10.50 ± 2.62	11.89 ± 3.51	< .001
Mayo stage III	23.42 ± 5.79	14.29 ± 3.72	13.27 ± 4.12	< .001
Mayo stage IV	23.86 ± 7.70	13.50 ± 4.02	14.67 ± 4.98	< .001

EBD = endoscopic biliary drainage, PTBD = percutaneous transhepatic biliary drainage.

## 4. Discussion

The preferred treatment for HCCA is radical surgical resection (R0 resection). However, this is often difficult to achieve. For patients for whom radical surgery is not possible, palliative surgery is performed. Some studies have shown that, even for advanced HCCA where R0 resection cannot be achieved due to positive margins, the survival rate of the surgical group is significantly higher than that of the nonsurgical group.^[12–14]^ A study by Jinmok Bae et al concluded that, despite margin resection status being an independent risk factor closely related to prognosis, palliative surgery can remove most lesions, reduce tumor load, and significantly improve survival rates.^[15]^ However, surgical treatment is more traumatic, and the complications caused by surgical trauma and stress cannot be ignored in patients in poor general condition or with poor nutritional status. Therefore, palliative surgery also needs to undergo strict evaluation and screening. In recent years, advanced drainage techniques such as PTBD and EBD have offered new treatment options for patients unable to undergo surgery. This study aimed to explore ways to select the most suitable treatment for different patients with intermediate to advanced HCCA.

There are currently 3 commonly used clinical staging systems for hepatocellular carcinoma: Bismuth-Corlette, Memorial Sloan Kettering Cancer Center, and Tumor, Node, Metastasis (TNM). Proposed by Bismuth et al in 1975, Bismuth–Corlette staging is based on the anatomical location of the tumor and does not consider other characteristics.^[16]^ It is primarily used for preoperative evaluation and surgical treatment planning and has limited prognostic value.^[17]^ The Memorial Sloan Kettering Cancer Center staging system is based on the Bismuth-Corlette system, to which an evaluation of portal vein invasion and hepatic atrophy has been added.^[18]^ Its primary purpose remains to define surgical resectability; however, it does not reliably indicate prognosis.^[19,20]^ First published by the American Joint Committee on Cancer in 1977, the TNM staging system is updated and revised every 6 to 8 years and has become the basis of clinical diagnosis and treatment. While it has a high predictive value for the prognosis of HCCA, it requires access to the pathological basis of the surgery and has some limitations in clinical use.^[21]^ Therefore, the Mayo staging system was developed. All of the parameters involved in the system can be obtained before surgery and can be applied to all patients. It has been demonstrated that the Mayo staging system is more effective than the TNM staging system for prognostic assessment in nonsurgical patients, providing better stratification.^[22]^ In this study, the Mayo staging system was therefore used to stratify HCCA patients and evaluate the safety and clinical efficacy of different yellowing reduction modalities in patients with HCCA and different Mayo stage classifications.

The results of this study show that, compared to preoperative levels, TBil, direct bilirubin, alanine aminotransferase, aspartate aminotransferase, gamma-glutamyl transferase, and alkaline phosphatase levels in the serum of the 3 groups all decreased significantly, indicating that all 3 methods can effectively drain and alleviate jaundice. For Mayo stages I and II, although palliative surgery requires a longer hospital stay, median survival is greater than for PTBD and EBD at 24 and 22.5 months, respectively. In terms of safety, there was no significant difference in the incidence of surgical complications compared to the PTBD and EBD groups. Therefore, patients with HCCA in Mayo stages I and II should prioritize palliative surgery. There was no statistical difference in median survival among the 3 groups of patients with Mayo stage III or IV. However, those who underwent palliative surgery experienced a more traumatic procedure, a slower recovery, and a longer hospital stay than those who underwent PTBD or EBD. Therefore, drainage techniques such as PTBD and EBD may be a more appropriate choice. EBD is more in line with physiological characteristics, does not require long-term catheterization in the later stages, and improves quality of life. Therefore, it is recommended for patients with generally good Mayo stage III conditions. However, the EBD group experienced a higher incidence of severe complications, such as acute pancreatitis and retrograde biliary tract infection, compared to the PTBD group. Among the Mayo stage IV patients in this study, 2 died from severe cholangitis and severe bleeding, respectively. Therefore, EBD requires a better overall physical condition than PTBD. For Mayo stage IV patients with poor general health, PTBD is more suitable. Additionally, some patients have difficulty undergoing EBD due to the high location of HCCA obstruction, as do patients with duodenal diverticula, nipple stenosis, and anatomical variations. For these patients, the PTBD method should also be used for drainage.^[23,24]^

PTBD and EBD are 2 techniques used to drain the biliary system. Both techniques have their respective advantages and disadvantages.^[25,26]^ Compared with the external drainage method of PTBD, EBD directs bile into the body’s natural physiological cavities, aligning more closely with their structure.^[27]^ EBD also prevents imbalances in water, electrolytes, and trace elements resulting from bile loss. It ensures normal digestive function after surgery and promotes patient recovery in the later stages.^[28]^ In this study, the incidence of electrolyte imbalance was significantly lower in the EBD group than in the PTBD group. However, EBD often requires duodenal papillary sphincterotomy, which carries a risk of perforating the digestive tract. There is also a risk of intestinal bacteria migrating retrograde into the bile duct and inducing cholangitis.^[29]^ In this study, the incidence of biliary tract infection was significantly higher in the EBD group than in the PTBD group. In addition, both the pancreatic duct and the common bile duct open into the duodenal papilla. EBD is more likely to cause acute pancreatitis, which explains why its incidence is higher in the EBD group than in the PTBD group. This finding is consistent with the results of previous research.^[30,31]^ PTBD is easier to perform, takes less time and has a higher success rate than EBD.^[32]^ However, there is a risk of accidentally injuring the intrahepatic blood vessels during PTBD puncture, which can cause biliary bleeding.^[8]^ In this study, biliary bleeding occurred in both the EBD and PTBD groups, which is consistent with previous research findings.^[33]^ This study found that the incidence of bile leakage was higher in the PTBD group than in the EBD group. This may be due to bile duct injury or incorrect placement of the drainage tube during the PTBD procedure.^[34]^ Furthermore, 1 patient in the PTBD group experienced drainage tube prolapse and required re-catheterization. Previous studies have also reported risks associated with PTBD, including needle tract transfer and drainage tube prolapse. Furthermore, using tubes for long periods affects patients’ quality of life in the later stages.^[7]^

Furthermore, the subgroup analysis based on preoperative TBil quartiles demonstrated a tiered efficacy profile. PTBD showed superiority in the Q1 subgroup, whereas palliative surgery provided optimal jaundice relief in the Q4 subgroup, potentially because procedures like bilio-enteric anastomosis create a more definitive and durable drainage pathway for severe obstructions. Consequently, preoperative TBil level should be a cornerstone in guiding personalized and precision treatment strategies.

To address concerns about selection bias in this retrospective study, we evaluated whether the observed survival benefit of palliative surgery was independent of the patients’ baseline condition. We also performed a multivariable Cox regression analysis. After adjusting for age, ECOG score, and Mayo stage, the treatment modality remained an independent predictor of survival. This provides compelling evidence that the survival advantage is more likely to be attributable to the method itself, potentially through the establishment of more definitive and durable biliary drainage, rather than to pretreatment patient characteristics. Although unmeasured confounding factors cannot be ruled out, our analysis strongly suggests that the treatment strategy is the primary determinant of patient outcome.

This is the first observational study to stratify HCCA patients using the Mayo staging system. Compared with other staging systems, the Mayo staging system is more effective at predicting survival periods. Furthermore, the Mayo staging system can be used for all HCCA patients, not just those who have undergone surgery. In addition to comparing the efficacy and safety of the 2 treatment methods (PTBD and EBD), this study also evaluated the efficacy and safety of palliative surgery. For patients who cannot undergo radical resection but whose survival can be prolonged by palliative resection, the study secured greater clinical benefits. Of course, this study has its limitations, too. Firstly, it is a retrospective, single-center study with a relatively small sample size. Further verification is required through multicenter, large-scale studies. Secondly, the study did not consider how factors such as the placement and depth of the drainage tube and stent, or other adjuvant treatments, might affect the results. Future research should consider these and other related factors to provide a more comprehensive analysis.

## 5. Conclusion

It can thus be concluded that for HCCA patients in Mayo stage I and II, palliative surgery can significantly prolong survival. Therefore, palliative surgical treatment should be given priority. For patients in Mayo stage III, as EBD is more in line with physiological characteristics and has a higher quality of life, it is recommended to adopt the EBD treatment approach. For patients with generally poor Mayo stage IV conditions and some who have difficulty undergoing EBD procedures, PTBD is a more appropriate choice.

In conclusion, when selecting a treatment plan for reducing jaundice in patients with intermediate and advanced HCCA, it is imperative to consider the tumor stage in conjunction with the merits and drawbacks of various jaundice treatment modalities. This comprehensive approach facilitates the formulation of an individualized treatment plan, with the objective of enhancing the treatment outcome and quality of life for the patient.

## Acknowledgments

The authors would like to thank all the reviewers who participated in the review

## Author contributions

**Conceptualization:** Zepu Wang.

**Data curation:** Zepu Wang.

**Formal analysis:** Zepu Wang.

**Funding acquisition:** Zepu Wang, Yanhui Peng.

**Investigation:** Dan Lv.

**Methodology:** Zepu Wang.

**Project administration:** Chuncheng Wang, Yanhui Peng.

**Resources:** Chuncheng Wang, Yanhui Peng.

**Software:** Dan Lv, Hanxiang Yu.

**Supervision:** Dan Lv, Hanxiang Yu.

**Validation:** Dan Lv, Hanxiang Yu.

**Visualization:** Hanxiang Yu.

**Writing** – **original draft:** Zepu Wang.

**Writing** – **review & editing:** Yanhui Peng.
